# Using Multistage Energy Barrier of Heterojunctions in Improving Cr(VI) Detection

**DOI:** 10.3390/ma16227154

**Published:** 2023-11-14

**Authors:** Minggang Zhao, Yichang He, Xiaotong Dong, Kun Pang, Qian He, Ye Ma, Hongzhi Cui

**Affiliations:** 1 School of Materials Science and Engineering, Ocean University of China, Qingdao 266100, China; 2 School of Chemistry and Chemical Engineering, Ocean University of China, Qingdao 266100, China

**Keywords:** CeO_2_, heterojunction, interface, Cr(VI), electrochemical

## Abstract

Detecting heavy metals in seawater is challenging due to the high salinity and complex composition, which cause strong interference. To address this issue, we propose using a multistage energy barrier as an electrochemical driver to generate electrochemical responses that can resist interference. The Ni-based heterojunction foams with different types of barriers were fabricated to detect Cr(VI), and the effects of the energy barriers on the electrochemical response were studied. The single-stage barrier can effectively drive the electrochemical response, and the multistage barrier is even more powerful in improving sensing performance. A prototype Ni/NiO/CeO_2_/Au/PANI foam with multistage barriers achieved a high sensitivity and recovery rate (93.63–104.79%) in detecting seawater while resisting interference. The use of multistage barriers as a driver to resist electrochemical interference is a promising approach.

## 1. Introduction

The pollution of seawater by heavy metals, including Cr(VI), is a significant and pressing issue [1,2,3]. Cr(VI) pollution, in particular, poses a severe threat to marine ecology, fisheries, and human health [4,5,6,7]. Thus, accurately detecting trace amounts of Cr(VI) in seawater is essential.

However, conventional methods are ineffective in seawater detection due to the strong interference caused by high salinity and complex composition. Various methods have been tried for the detection of Cr(VI), including fluorescence probe, colorimetry, and so on [8,9], but the reliable methods used in seawater are still the traditional atomic absorption spectrometry and plasma mass spectrometry [10,11]. Although these methods are accurate, there are deficiencies that limit their application, such as their high equipment cost, time-consuming process, and complex technical requirements. The rapid online Cr(VI) detection in seawater remains a challenge. Electrochemical sensors have significant advantages in online detection due to their rapid response, high sensitivity, and simple operation. Since most electrochemical tests are performed on a redox basis, it is difficult to avoid interference from redox reactions in the test system. This shortcoming limits its application in complex water environments [12].

Semiconductor heterojunctions have been widely applied in fields such as photoelectric materials, sensors, batteries, and more, as they can effectively regulate electron transport through their interfacial effect [13,14,15]. For instance, the CuO/In_2_O_3_ p-n junction interface has been shown to promote the catalytic process of dimethyldichlorosilane [16], while Rh/ZnO Schottky contacts have been found to improve the asymmetric rectification properties of materials [17]. Special heterojunction interfaces can form interfacial barriers that hinder electron transport, and changes in these barriers can affect resistance [18]. In the context of electrochemical sensing, changes in impedance can affect electrochemical current response. As a result, barriers hold promise as an electrochemical sensing driver to generate electrochemical response. Moreover, as barriers are primarily changed by physical factors independent of redox reactions, electrochemical interference is expected to be largely suppressed.

Two typical barriers that can be formed are p-n junctions and Schottky junctions. NiO (a p-type semiconductor) and CeO_2_ (an n-type semiconductor) can form a p-n junction [19]. Au, with a work function of 5.1 eV, can form Schottky junctions with NiO and CeO_2_, respectively [20,21,22]. The matching energy band structures enable them to form the NiO/CeO_2_/Au heterojunction with multistage barriers. Polyaniline (PANI) has abundant amine and imine groups, which allow it to selectively adsorb Cr(VI) [23].

Herein, the Ni/NiO/CeO_2_/Au/PANI foam was prepared for direct Cr(VI) detection. A multistage barrier was used as an electrochemical driver to generate an electrochemical current response to Cr(VI) by combining it with a selective adsorption film. Different types of barriers were investigated, and the sensing performance was improved significantly by using multistage barriers. The trace detection of Cr(VI) with a high recovery rate (93.63–104.79%) in seawater was achieved by resisting the strong interference of seawater. Using a multistage barrier as an electrochemical driver is an effective approach to resist interference.

## 2. Experimental

### 2.1. Reagents

All the reagents used in this research were of analytical grade. Uric acid, xylene, aspartic acid, hydroquinone, p-phenylenediamine, KCl, NaCl, AgCl, Zn(NO_3_)_2_·6H_2_O, CoSO_4_·7H_2_O, CuSO_4_·7H_2_O, SnCl_2_·2H_2_O, Bi(NO_3_)_3_·5H_2_O, Fe(NO_3_)_3_·9H_2_O, and K_2_Cr_2_O_7_ were all purchased from Aladdin Reagent (Shanghai, China) Co., LTD. Polyvinylpyrrolidone (PVP), hemoglobin (Hb), and dopamine (DA) were purchased from Alfa Aesar. Ce(NO_3_)_3_·6H_2_O, acetone, ethanol, and hydrochloric acid (HCl) were all bought from Sinopharm Chemical Reagents Co., LTD. (Shanghai, China). Ni foam was purchased from Hengxin Research Metal Co., LTD. (Baoji, China). All of the water used was deionized water.

### 2.2. Equipment and Measurements

All electrochemical experiments were performed using the electrochemical workstation (CHI760E, Chenhua, Shanghai, China). Scanning electron microscopy (SEM) images were captured using an S-4800 SEM equipment (Tokyo, Japan). X-ray diffraction (XRD) analysis was conducted using an Ultima IV X-ray diffractometer (Tokyo, Japan) (2θ = 10–80°, scan velocity 10°/min, Cu Kα radiation). Infrared spectroscopy data were collected using the Nicolet iS10 Fourier Transform Infrared (FTIR) spectrometer (Waltham, MA, USA).

During the FTIR test in a dry environment, the attenuated total reflectance (ATR) accessory was placed in the optical path of the spectrometer, and the air background was scanned. The surface of the bulk sample (1 cm wide and 1 cm long) was snared to the crystal surface of the ATR accessory. The infrared spectrum of the sample was collected with a resolution of 4 cm^−1^. Scanning was performed 32 times.

In the XRD test, the sample was cut into a small piece with a width and length of 1cm to ensure that the test surface was smooth, and the sample was fixed on the sample table with plasticine. Then, the XRD test was started.

The SEM test involved directly gluing the sample to the conductive adhesive and using the scanning electron microscope to photograph the sample morphology and energy spectrum mapping.

### 2.3. Preparation of the Ni/NiO/CeO_2_/Au/PANI Foam

The Ni foam (1 × 1.5 cm) was first cleaned sequentially in propanone, ethanol, and deionized water using ultrasonic cleaning for 15 min each. After drying with nitrogen, it was transferred to a tube furnace and heated to 700 °C within 300 min; then, it was held at that temperature for 240 min. The Ni/NiO foam was obtained after cooling to room temperature.

Furthermore, 0.185 g of Ce(NO_3_)_3_·6H_2_O and 0.06 g of PVP were dissolved in 50 mL of deionized water and stirred at ambient temperature for 30 min. The resulting mixture was then transferred into a 100 mL TEFLON reactor. Ni/NiO foam was added and subjected to a hydrothermal reaction at 180 °C for 12 h. After cooling, the sample was removed, and the surface impurities were cleaned using ethanol and deionized water. The Ni/NiO/CeO_2_ foam was obtained after drying at 60 °C.

Au was sputter-coated on the Ni/NiO/CeO_2_ foam for 90 s on both sides to obtain Ni/NiO/CeO_2_/Au foam. PANI film was deposited onto the surface of the prepared Ni/NiO/CeO_2_/Au foam via the three-electrode potentiostatic method. The electrodeposition was carried out at a voltage of 1.2 V using 0.5 M HCl as electrolyte with 0.3 M aniline monomer at 35–40 °C. The Ni/NiO/CeO_2_/Au/PANI foam was obtained after cleaning and vacuum drying at 40 °C for 6 h.

### 2.4. Electrochemical Test

A three-electrode system was used for electrochemical testing, with the Ni/NiO/CeO_2_/Au/PANI foam serving as the working electrode, a platinum electrode as the counter electrode, and an Ag/AgCl electrode as the reference electrode. Phosphate-buffer solution (PBS) with a pH of 5.8 was used as the electrolyte.

## 3. Results and Discussion

### 3.1. Characterization of the Ni/NiO/CeO_2_/Au/PANI Foam

**Figure 1:**
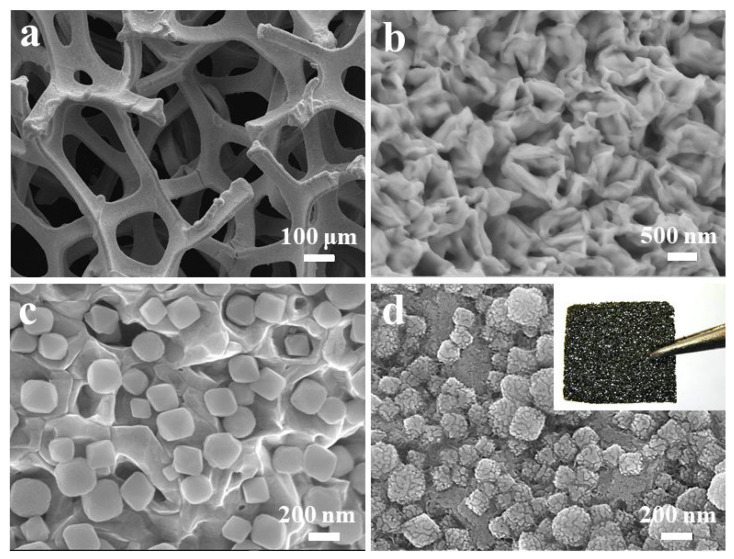
SEM images of (**a**) the Ni/NiO foam, (**b**) locally amplified surface of the Ni/NiO foam, (**c**) locally amplified surface of the Ni/NiO/CeO_2_ foam, and (**d**) locally amplified surface of the Ni/NiO/CeO_2_/Au/PANI foam. The inset is a photograph of the Ni/NiO/CeO_2_/Au/PANI foam.

The synthesis process of the Ni/NiO/CeO_2_/Au foam is presented in Figure S1, which involves a layered assembly process. Initially, a NiO nanolayer was thermally oxidized on the surface of the Ni foam. Next, a CeO_2_ nanolayer was deposited on the NiO surface but not entirely covering it. Afterward, the Ni/NiO/CeO_2_ foam was sputter-coated with Au nanoparticles to form the Ni/NiO/CeO_2_/Au foam. The SEM images in Figure 1 show that the Ni/NiO foam has a three-dimensional porous framework (Figure 1a) with numerous NiO nanofolds distributed on its surface (Figure 1b). The CeO_2_ cubes, with an edge length of approximately 200–300 nm, are uniformly distributed on the NiO nanofolds but not completely covering them (Figure 1c). As a result, the sprayed Au nanoparticles are in contact with both NiO and CeO_2_. Finally, Figure 1d presents a photo of the Ni/NiO/CeO_2_/Au/PANI foam with a porous 3D frame structure. The pleated PANI film, with a thickness of about dozens of nanometers, uniformly covers the Ni/NiO/CeO_2_/Au foam after electrodeposition. The EDS images show that N, Ni, O, Ce, Au, and C elements are uniformly distributed on the Ni/NiO/CeO_2_/Au/PANI foam (Figure S2).

**Figure 2:**
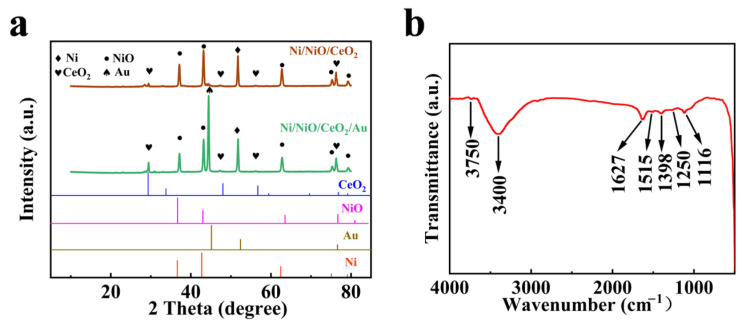
(**a**) XRD patterns of the Ni/NiO/CeO_2_ foam and the Ni/NiO/CeO_2_/Au foam. (**b**) FTIR spectra of the Ni/NiO/CeO_2_/Au/PANI foam.

The XRD patterns of the Ni/NiO/CeO_2_ foam and the Ni/NiO/CeO_2_/Au foam are shown in Figure 2a. The diffraction peak at 51.8° corresponds to the (200) crystal plane of Ni, while the diffraction peaks at 37.2°, 43.3°, 62.9°, 75.4°, and 79.4° correspond to the (111), (200), (220), (311), and (222) crystal planes of NiO, respectively. The diffraction peaks at 28.6°, 47.5°, 56.3°, and 76.7° correspond to the (111), (220), (311), and (331) crystal planes of CeO_2_, respectively [24]. The diffraction peak at 44.4° corresponds to the (200) crystal planes of Au. There are no impurity peaks in the XRD patterns, indicating that the samples were successfully prepared.

The FTIR spectra of the Ni/NiO/CeO_2_/Au/PANI foam are shown in Figure 2b. The absorption peak at 1116 cm^−1^ and 1398 cm^−1^ are related to the C-H bond vibration of the aromatic ring and the C-N stretching vibration of the aromatic amine, respectively. The absorption peak at 1250 cm^−1^ corresponds to the C-N^+^ stretching vibration in the polaron structure [25]. The absorption peaks at 1515 cm^−1^ and 1627 cm^−1^ are caused by the stretching vibration of the C=C bonds of quinone and benzene rings separately, while the absorption peaks at 3400 cm^−1^ are mainly caused by the stretching vibration of N-H bonds in PANI [26,27]. The absorption peak at 3750 cm^−1^ corresponds to the vibration of the free O-H group [28]. The existence of multiple functional groups in the FTIR spectra further confirms that PANI film was successfully deposited on the Ni/NiO/CeO_2_/Au foam.

### 3.2. Detection Mechanism

The barrier (Φ) acts as a hindrance to electron transport, and any change in its height (ΔΦ) can affect the electrochemical current response by altering the impedance. The equipotential point for Hb is at pH = 7.4, and when the pH is 5.8, the ion is positively charged [29]. This can create electrostatic induction, leading to a change in ΔΦ by modifying the energy bands of the heterojunction. Hence, the barrier effects on the electrochemical current response can be studied by utilizing the electrostatic induction of Hb.

**Figure 3:**
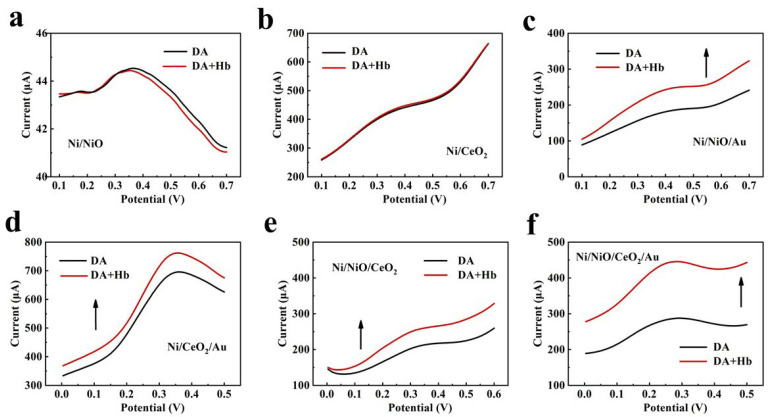
The electrochemical current response to DA is used as a reference basis; DPV curves of the prepared foams in the presence and absence of 0.5 mg/mL positively charged Hb in PBS, respectively. (**a**) The Ni/NiO foam, (**b**) the Ni/CeO_2_ foam, (**c**) the Ni/NiO/Au foam, (**d**) the Ni/CeO_2_/Au foam, (**e**) the Ni/NiO/CeO_2_ foam, (**f**) the Ni/NiO/CeO_2_/Au foam.

The electrochemical current response to DA was used as a reference basis, and the results are shown in Figure 3. For the Ni/NiO foam and the Ni/CeO_2_ foam without an interfacial barrier (Figure 3a,b), the electrochemical current response changed slightly with the addition of Hb. However, for the Ni/NiO/Au foam, Ni/CeO_2_/Au foam, Ni/NiO/CeO_2_ foam, and Ni/NiO/CeO_2_/Au foam with an interfacial barrier, the electrochemical current response increased significantly with the addition of Hb (Figure 3c–f). These results indicate that the barrier can indeed be used as an electrochemical sensing driver to generate an electrochemical current response. A further comparison found that the prepared foams with a Schottky barrier (Ni/NiO/Au foam, Ni/CeO_2_/Au foam) produced a larger response than the prepared foam with a p-n junction barrier (Ni/NiO/CeO_2_ foam), and the foam with a multistage barrier (Ni/NiO/CeO_2_/Au foam) had the most significant response.

**Figure 4:**
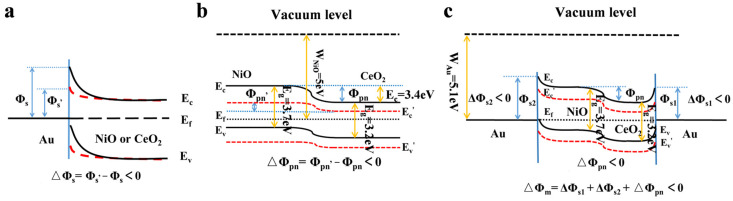
The energy band diagram of the prepared foams with (**a**) Schottky barrier, (**b**) p-n junction barrier, and (**c**) multistage barrier. Electrostatic induction of Hb causes the height of the three kinds of barrier to reduce.

The above results can be explained by referring to the energy band diagram presented in Figure 4 and Figure S3. For the Ni/NiO/Au foam and the Ni/CeO_2_/Au foam with a Schottky barrier (Figure 4a), the positively charged Hb induces negative charges in the semiconductor conduction band of NiO or CeO_2_ on one side, resulting in a decrease in barrier height (△Φ_s_ < 0), a decrease in impedance, and an increase in current response. In contrast, for the Ni/NiO/CeO_2_ foam with a p-n junction barrier (Figure 4b), since CeO_2_ does not completely cover the NiO nanolayer, both the conduction bands of NiO and CeO_2_ decrease due to the electrostatic induction of Hb. In this case, the p-n junction barrier height changes as △Φ_pn_ = Φ_pn_′ − Φ_pn_ < 0 [30], leading to an increase in electrochemical current response. Compared to the Schottky junction, where the conduction band lowers on only one side, the decrease in the conduction bands on both sides of the p-n junction weakens the reduction in barrier height, namely, |△Φ_s_| > |△Φ_pn_|. Hence, the foam with a p-n junction barrier exhibits a smaller change in response than the foam with a Schottky barrier. In the case of the Ni/NiO/CeO_2_/Au foam with a multistage barrier (Figure 4c), three barriers, including two Schottky barriers (NiO/Au and CeO_2_/Au) and one p-n junction barrier (NiO/CeO_2_), are present. The electrostatic induction reduces the height of all three barriers simultaneously (△Φ_m_ = △Φ_s1_ + △Φ_s2_ + △Φ_pn_), leading to the largest change in electrochemical current response. The above results demonstrate that the barrier can be employed as an electrochemical driver to generate an electrochemical response, and the performance can be enhanced by employing a multistage barrier.

**Figure 5:**
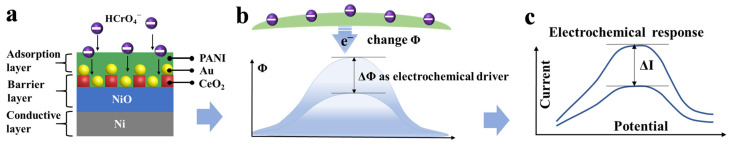
The proposed sensing mechanism for Cr(VI) detection. (**a**) Schematic diagram of the Ni/NiO/CeO_2_/Au/PANI foam for adsorption of HCrO_4_^−^, (**b**) the formed negative-charge layer causes the height of barrier change, (**c**) the changed barrier drives electrochemical current response.

The form of Cr(VI) in solution is dependent on the pH value, existing as HCrO_4_^−^ at 1 < pH < 6.8 [31]. Under acidic conditions, PANI exhibits specific adsorption to HCrO_4_^−^ [32]. The proposed sensing mechanism is illustrated in Figure 5. The fabricated foam electrode comprises three functional layers, namely, the selective adsorption layer, barrier layer, and conductive layer (Figure 5a). The outer PANI film selectively adsorbs HCrO_4_^−^ to form a negatively charged layer, which obtains holes from the semiconductor (analogous to the semiconductor gaining electrons) to maintain the charge balance. The conduction bands of the semiconductors reduce as they gain electrons from the negatively charged layer, leading to changes in barrier height, which generate the driving force for the electrochemical current response (Figure 5b,c). Thus, Cr(VI) detection is achieved through the use of a barrier as an electrochemical driver combined with specific adsorption. The barrier serves as the driver, and different barrier layers can achieve distinct sensing performance.

### 3.3. Electrochemical Detection

**Figure 6:**
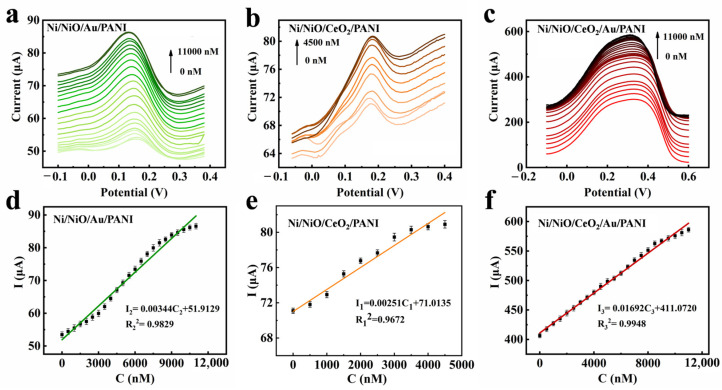
DPV curves of the prepared foams for various Cr(VI) concentrations in 0.1 M PBS at pH = 5.8. (**a**) The Ni/NiO/Au/PANI foam with Schottky barrier, (**b**) the Ni/NiO/CeO_2_/PANI foam with p-n junction barrier, (**c**) the Ni/NiO/CeO_2_/Au/PANI foam with multistage barrier. The electrochemical current response increases with increased Cr(VI) concentrations. (**d**–**f**) The corresponding calibration curve of Cr(VI) concentration versus current.

Based on the above analysis, electrochemical tests were performed on Ni/NiO/CeO_2_/Au/PANI foams at different pH values, and the optimal pH for the reaction was obtained through experimental evidence (Figure S4). The foams with different barriers were tested for Cr(VI) detection in PBS at pH = 5.8. The results in Figure 6 demonstrate that all the prepared foams can achieve linear detection of Cr(VI), with the electrochemical current response increasing proportionally to the concentration of Cr(VI). The detection performance of the foams is summarized as follows:

The Ni/NiO/CeO_2_/PANI foam with a p-n junction barrier: the sensitivity is 2.47 × 10^−3^ μA·nm^−1^·cm^−2^. The linear regression equation is I = 0.00247C + 71.1343 (R = 0.9619) (0–4500 nM). The lowest detection limit (LOD) is 244.43 nM. The Ni/NiO/Au/PANI foam with a Schottky barrier: the sensitivity is 3.44 × 10^−3^ μA·nm^−1^·cm^−2^. The linear regression equation is I = 0.00344C + 51.9129 (R = 0.9829) (0–11,000 nM). LOD is 175.51 nM. The Ni/NiO/CeO_2_/Au/PANI foam with a multistage barrier: the sensitivity is 16.92 × 10^−3^ μA·nm^−1^·cm^−2^. The linear regression equation is I = 0.01692C + 411.0720 (R = 0.9948) (0–11,000 nM). LOD is 35.68 nM. From the above results, it can be found that the order of Cr(VI) detection performance is as follows: the Ni/NiO/CeO_2_/PANI foam < the Ni/NiO/Au/PANI foam < the Ni/NiO/CeO_2_/Au/PANI foam. Compared with much of the previous work presented in Table 1, the Ni/NiO/CeO_2_/Au/PANI foam is able to maintain a lower detection limit while detecting a wide concentration range.

**Figure 7:**
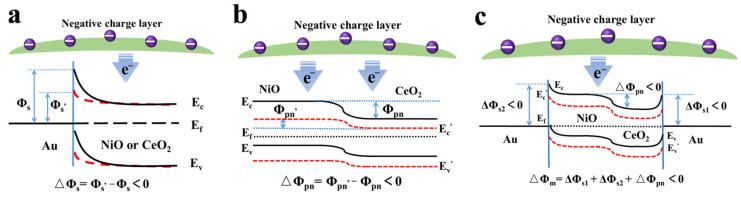
The energy band diagram of the prepared foams with (**a**) Schottky barrier, (**b**) p-n junction barrier, and (**c**) multistage barrier. The negatively charged layer formed through HCrO_4_^−^ adsorption causes the height of the three kinds of barrier to reduce.

The sensing mechanism is further discussed in Figure 7. The outer PANI film specifically adsorbs HCrO_4_^−^ to form a negatively charged layer. For the Ni/NiO/Au/PANI foam, NiO gains electrons from the layer, and the conduction band reduces. Therefore, the height of the Schottky barrier decreases, and the current response increases (△Φ_S_ < 0). For the Ni/NiO/CeO_2_/PANI foam, NiO and CeO_2_ gain electrons from the negatively charged layer, and their conduction bands reduce. In this case, the height of the p-n junction barrier also decreases (△Φ_pn_ < 0) [30], resulting in the increased electrochemical current. Since the band of the Schottky barrier reduces on one side, |△Φ_S_| > |△Φ_pn_ |. Therefore, the sensing performance of the Ni/NiO/Au/PANI foam is better than that of the Ni/NiO/CeO_2_/PANI foam. For the Ni/NiO/CeO_2_/Au/PANI foam, NiO and CeO_2_ gain electrons from the negatively charged layer, and their conduction bands reduce. In this case, the height of two Schottky barriers (NiO/Au, CeO_2_/Au) and one p-n junction barrier (NiO/CeO_2_) decreases simultaneously (△Φ_m_ = △Φ_s1_ + △Φ_s2_ + △Φ_pn_), so the electrochemical current increases even more. The above theoretical analysis is consistent with the DPV results and the electrochemical impedance spectroscopy test (Figure S5).

In addition, the controls without interfacial barriers (the Ni/NiO/PANI and the Ni/CeO_2_/PANI foam) were also tested in Cr(VI) detection. As shown in Figure S6, no effective electrochemical current response is found with the increased HCrO_4_^−^ concentration, indicating that the interfacial barrier is the electrochemical driver to generate electrochemical response.

**Figure 8:**
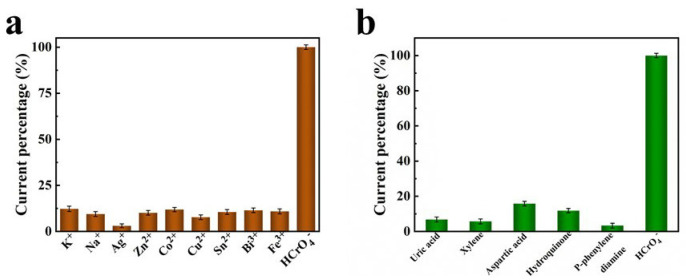
The selectivity of the Ni/NiO/CeO_2_/Au/PANI foam to HCrO_4_^−^ in the presence of other interfering substances with the same concentration (2000 nM), including (**a**) common metal ions and (**b**) organic pollutants in seawater.

The detection of Cr(VI) in seawater can be affected by various metal ions and organic pollutants, making it crucial to ensure interference resistance. As depicted in Figure 8a,b, the Ni/NiO/CeO_2_/Au/PANI foam exhibits a significantly higher response to HCrO_4_^−^ than other interfering substances with the same concentration, such as common metal ions and organic pollutants found in seawater. These results demonstrate that the Ni/NiO/CeO_2_/Au/PANI foam is capable of resisting common interferences in seawater.

Real-life samples from tap water and seawater were examined, and the results are shown in Table 2. The recoveries of the tap water samples range from 94.72% to 106.09% with relative standard deviations (RSD) of 2.53–5.59%. The recoveries of seawater are 93.63–104.79% with RSD of 1.58–5.40%. These results demonstrate that the Ni/NiO/CeO_2_/Au/PANI foam is capable of resisting common interferences in seawater.

The resistance to interference in seawater is mainly attributed to the following factors. Firstly, the multistage barrier is used as the electrochemical driver to generate the electrochemical response, which is different from the traditional electrochemical response based on a redox reaction. This sensing mode relies on physical electrostatic induction, which shields the interference of many chemically active substances. In addition, the electrochemical current response produced by changing the barrier takes the form of exponential amplification: J∝-exp(Φ/2k_0_T) [29]; thus, other interference signals are suppressed correspondingly. Thirdly, other interfering metal ions cannot be absorbed by the PANI film to generate electrostatic induction, which means they cannot change the barrier to generate an electrochemical response.

**Figure 9:**
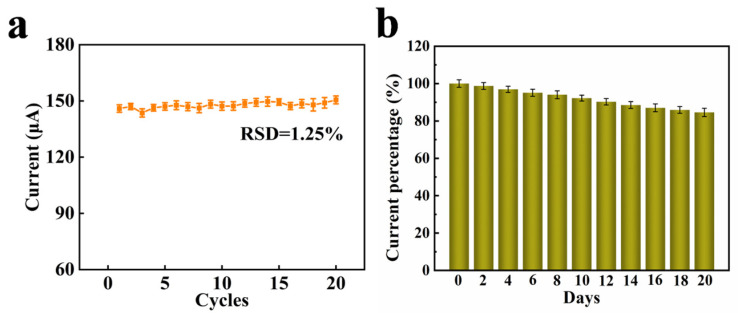
(**a**) Short-term stability of the Ni/NiO/CeO_2_/Au/PANI foam from 20 consecutive tests. (**b**) Long-term stability of the Ni/NiO/CeO_2_/Au/PANI foam with the addition of 2000 nM HCrO_4_^−^.

The stability of the Ni/NiO/CeO_2_/Au/PANI foam was studied using the cyclic electrode method. As shown in Figure 9a, no significant change in current response can be observed after 20 consecutive tests, and the RSD is 1.13%, indicating good short-term stability. In addition, the DPV response to HCrO_4_^−^ is measured every 2 days for 20 days. The final electrochemical response remains above 80% (Figure 9b), indicating good long-term stability.

## 4. Conclusions

The Ni based foams with different interfacial energy barriers were prepared for Cr(VI) detection. It was found that a barrier can be used as an electrochemical driver to generate an electrochemical response. The electrostatic interaction of Cr(VI) regulates the energy barrier without causing redox and improves the specificity in seawater. Different types of barriers have different levels of performance. Compared with the traditional single-stage barriers, the multistage barriers designed in this experiment can improve the sensing sensitivity by 5–7 times. The fabricated Ni/NiO/CeO_2_/Au/PANI foam achieved the highly sensitive detection of Cr(VI) by resisting the strong interference of seawater. The obtained outstanding LOD (35.68 nM) and recovery (93.63–104.79%) make it promising for practical applications. The development of electrochemical sensors using multistage barriers as electrochemical drivers to resist interference represents a new approach.

## Figures and Tables

**Table 1:** Comparison of the sensing performance of various sensors to Cr(Ⅵ).

Electrode Materials	Method	LOD (nM)	Linear Ranges (μM)	Ref
Single-atom Fe catalysts	Colorimetric	3.00	0.0300–3.00	[33]
Cu-PyC	Colorimetric	51.0	0.500–50.0	[34]
CTAB-MoS_2_/rGO	Colorimetric	1.25	0.0100–10.0	[35]
Au NDC@Ag NRs	Colorimetric	1.69 × 10^3^	2.50–40.0	[36]
Cr(VI)	Colorimetric	100	0.200–50.0	[37]
g-C_3_N_4_/AgM/Nf/GCE	Electrochemistry	1.60	0.100–0.700	[32]
GCE/PSF^+^-MS-SO_3_^−^	Electrochemistry	500	1.00–1.00 × 10^2^	[38]
Ni/NiO/CeO_2_/Au/PANI	Electrochemistry	35.7	0.500–11.0	This work

**Table 2:** Detection of Cr(Ⅵ) in real environment by using the Ni/NiO/CeO_2_/Au/PANI foam.

		Tapwater			Seawater	
Added (nM)	Found (nM)	Recovery (%)	RSD (%)	Found (nM)	Recovery (%)	RSD (%)
1000	1060.87	106.09	3.62	936.30	93.63	5.40
2000	1984.36	94.72	2.53	1880.06	94.00	1.66
3000	2937.46	97.92	5.59	3143.90	104.79	2.40
4000	4043.72	101.09	4.29	3853.69	96.34	1.58

## Data Availability

The data presented in this study are available on request from the corresponding author. The data are not publicly available due to privacy.

## Supplementary Materials

The following supporting information can be downloaded at: https://www.mdpi.com/article/10.3390/ma16227154/s1, Figure S1. The synthesis process of the Ni/NiO/CeO_2_/Au foam. Figure S2. EDS mapping of N, Ni, O, Ce, Au and C on the Ni/NiO/CeO_2_/Au/PANI foam. Figure S3. The actual band positions with (a) NiO/CeO_2_ and (b) NiO/CeO_2_/Au. Figure S4. Effect of pH value on Cr (VI). Reaction conditions: pH = 1–11, 2 μM Cr (VI), 0.1 M PBS and 25 ℃. The maximum point in curve was set as 100%. Figure S5. The electrochemical impedance spectroscopy of the Ni/NiO/CeO_2_/PANI foam (a) and the Ni/NiO/CeO_2_/Au/PANI foam (b) in presence and absence of HCrO_4_^−^. Figure S6. DPV curves of the Ni/NiO/PANI foam (a) and the Ni/CeO_2_/PANI foam (b) for various C(VI) concentrations in 0.1 M PBS at pH = 5.8.
