# Supplementary material for: Using Multistage Energy Barrier of Heterojunctions in Improving Cr(VI) Detection
Source: Materials (Basel). 2023 Nov 14;16(22):7154. doi: 10.3390/ma16227154 (PMC10672457; doi:10.3390/ma16227154)
Supplement: Supplementary file 1 [file materials-16-07154-s001.zip › materials-2696843-supplementary.pdf]

Supplementary material

Using multistage energy barrier of heterojunctions in improving Cr(VI) detection

Minggang Zhao[a,1], Yichang He[a,1], Xiaotong Dong[a,1], Qian He[b], Ye Ma[a]*, Hongzhi Cui[a]*

[a]School of Materials Science and Engineering, Ocean University of China, 266100, Qingdao, P. R. China

[b]School of Chemistry and Chemical Engineering, Ocean University of China, 266100, Qingdao, P. R. China

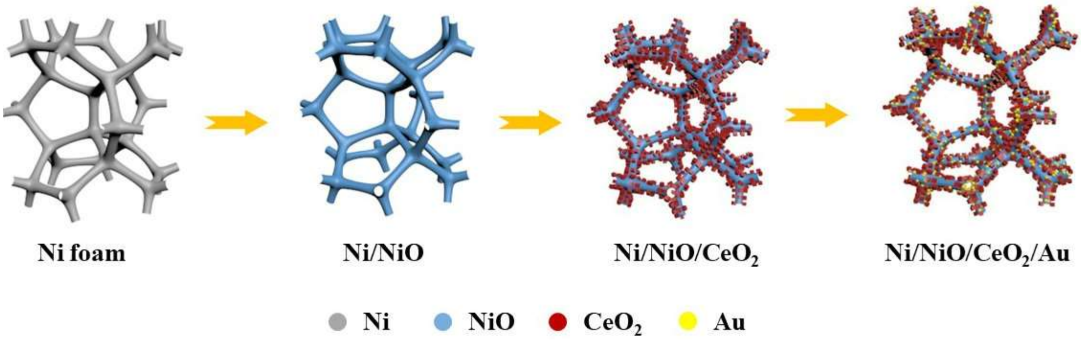

Figure S1. The synthesis process of the Ni/NiO/CeO_2/Au foam.

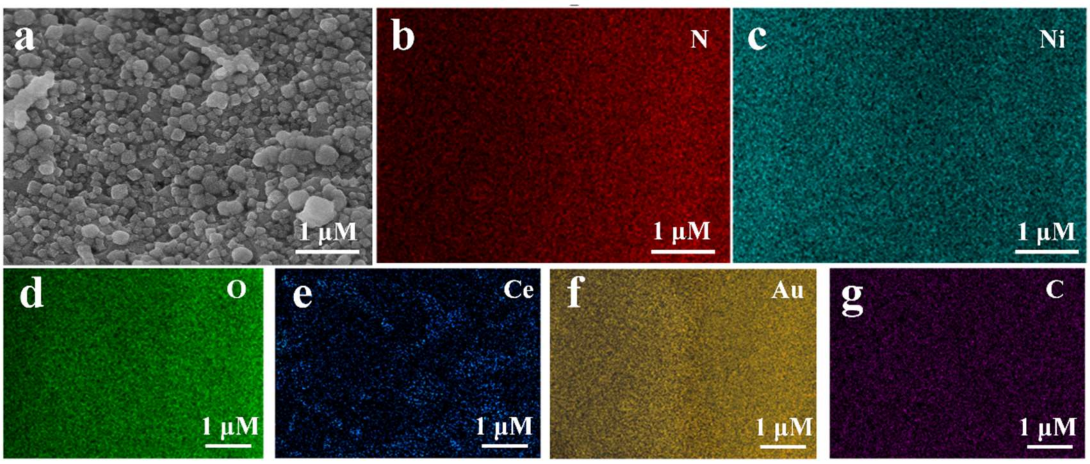

Figure S2. EDS mapping of N, Ni, O, Ce, Au and C on the Ni/NiO/CeO_2/Au/PANI foam.

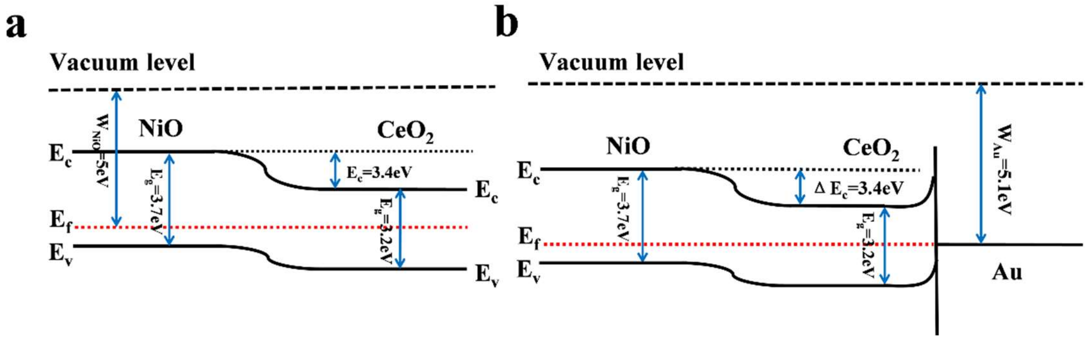

Figure S3. The actual band positions with (a)NiO/CeO_2 and (b) NiO/CeO_2/Au.

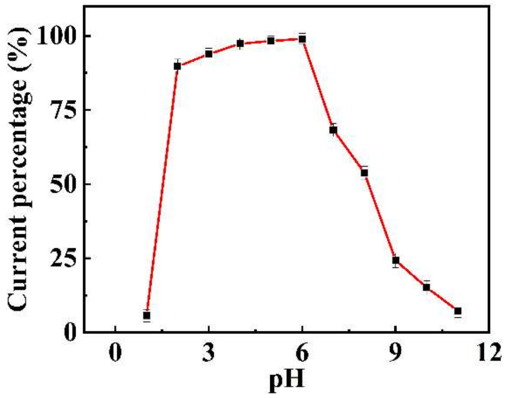

Figure S4. Effect of pH value on Cr (VI). Reaction conditions: pH=1-11, 2μM Cr (VI), 0.1M PBS and 25°C. The maximum point in curve was set as 100 %.

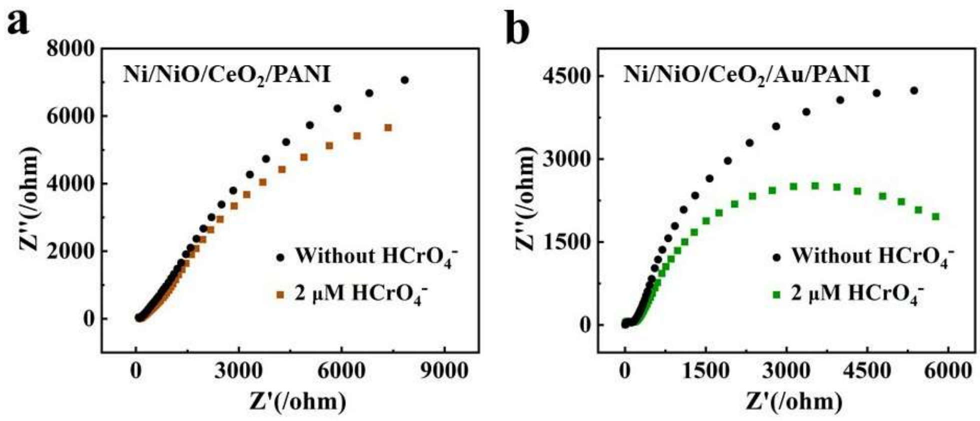

Figure S5. The electrochemical impedance spectroscopy of the Ni/NiO/CeO_2/PANI foam (a) and the Ni/NiO/CeO_2/Au/PANI foam (b) in presence and absence of $HCrO_4^-$.

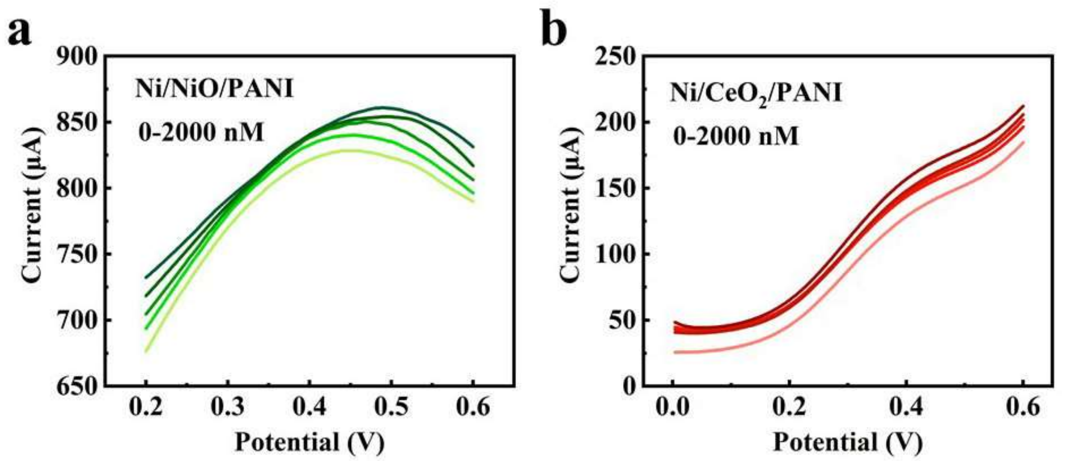

Figure S6. DPV curves of the Ni/NiO/PANI foam (a) and the Ni/CeO_2/PANI foam (b) for various C(VI) concentrations in 0.1 M PBS at pH=5.8.
